# A rare occurrence of paragonimiasis accompanied by cryptococcal pneumonia

**DOI:** 10.1002/rcr2.1424

**Published:** 2024-07-19

**Authors:** Hwan Jin Lee, Jun Hyung Park, Ae Ri Ahn, Jae Seok Jeong, Yong Chul Lee

**Affiliations:** ^1^ Department of Internal Medicine, Research Center for Pulmonary Disorders Jeonbuk National University Medical School Jeonju Republic of Korea; ^2^ Research Institute of Clinical Medicine of Jeonbuk National University‐Biomedical Research Institute of Jeonbuk National University Hospital Jeonju Republic of Korea; ^3^ Department of Pathology Jeonbuk National University Medical School Jeonju Republic of Korea; ^4^ Respiratory Drug Development Research Institute Jeonbuk National University Medical School Jeonju Republic of Korea; ^5^ Laboratory of Respiratory Immunology and Infectious Diseases, Korea Zoonosis Research Institute Jeonbuk National University Iksan Republic of Korea

**Keywords:** cryptococcal infection, *Paragonimus westermani*, pulmonary paragonimiasis

## Abstract

Pulmonary paragonimiasis may be accompanied by a rare infectious disease, such as cryptococcal pneumonia. To our knowledge, this is the first case ever reported in the English literature.

## Graphical abstract

To our knowledge, this is the first case ever reported in the English literature of pulmonary paragonimiasis accompanied by cryptococcal pneumonia.

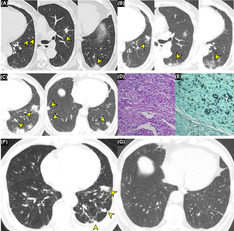

## CLINICAL IMAGE

**FIGURE 1:**
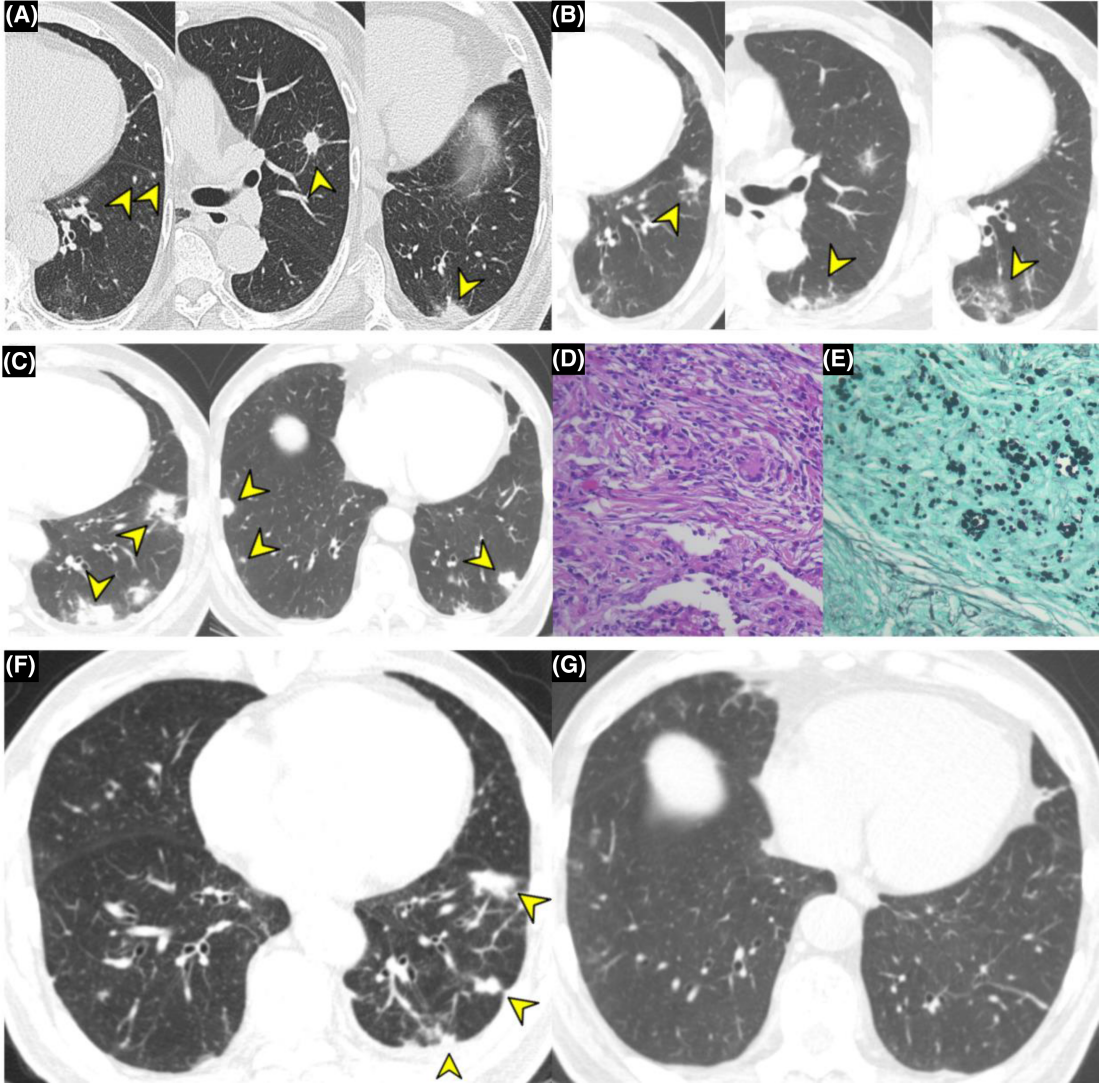
(A) Initial high resolution computed tomography (HRCT) shows nodular consolidation measuring 1.5 cm with spiculated margin in the lingular segment (arrow) and multiple variable sized pulmonary nodules (arrow) in left lung. (B) Three weeks after treatment for PW, previous pulmonary nodule in left upper lobe markedly decreased on non‐enhanced chest CT. However, previous pulmonary nodule near left major fissure increased (arrow), and new multiple subpleural nodules with diffuse ground glass opacity including bronchiectasis in superior segment of LLL (arrow) are shown on HRCT. (C) Ten weeks later from initial HRCT, followup non‐enhanced chest CT shows increased size of previous wedge‐shaped subpleural nodules in left lower lobe and multiple new subpleural nodules in both lower lobes, suggesting cryptococcal infection (arrows). (D) Histopathological findings revealed granulomatous inflammation composed of multinuclear giant cell, some lymphocytes, and plasma cells (haematoxylin–eosin [H&E], 400×). (E) Grocott‐methenamine silver (GMS) stain showed positivity of typical characteristic encapsulated yeast cells with budding, confirming pulmonary cryptococcal infection (original magnification, 400×). (F) After the initial three‐week treatment with fluconazole, the focal subfissural consolidation in LLL decreased significantly (arrows). (G) Followup with HRCT imaging after 3 months shows that multiple solid nodules markedly improved in both lung parenchyma.

A male in his 80s presented with pulmonary nodules. He had never smoked and had diabetes in an uncontrolled state with a haemoglobin A1C of 7.9%. Additionally, anti‐HIV antibody was negative. Physical examination was unremarkable and laboratory data revealed peripheral eosinophilia as 810/uL. High‐resolution computed tomography (HRCT) showed multiple pulmonary nodules in left lung (Figure 1A, arrows). Along with a positive result on enzyme‐linked immunosorbent assay against *Paragonimus westermani* and image findings, the patient was given 25 mg/kg of praziquantel for 2 days. The drug of choice for paragonimiasis is praziquantel 25 mg/kg three times per day for two consecutive days with a high efficacy of approximately 90%.^1^ Therefore, no additional medication was administered. Five weeks later, previous pulmonary nodules in left upper lobe markedly decreased. However, the previous pulmonary nodule near the left major fissure increased and new multiple nodules were seen on HRCT (Figure 1B, arrows). Ten weeks later, follow‐up non‐enhanced chest CT showed increased size of previous wedge‐shaped subpleural nodules in the left lower lobe and multiple new subpleural nodules in both lower lobes. The presence of lesions in the lower periphery of the lung and the existence of halo signs led to the suspicion of a fungal infection (Figure 1C, arrows).^2^ Radiologist ruled out tuberculosis based on the image findings and several tests for tuberculosis such as bronchial washing and polymerase chain reaction assay showed negative results. To confirm the lesion, surgical resection was performed and histopathology showed granulomatous inflammation (Figure 1D). Positive grocott‐methenamine silver stain confirmed cryptococcal infection (Figure 1E). After initial treatment with fluconazole, focal subfissural consolidation in LLL decreased significantly (Figure 1F, arrows). After taking fungal agent, most pulmonary lesions markedly improved (Figure 1G). Our case highlights that pulmonary paragonimiasis may be accompanied by a rare infectious disease, such as cryptococcal pneumonia. To our knowledge, this is the first case ever reported in the English literature.

## AUTHOR CONTRIBUTIONS

Hwan Jin Lee and Jun Hyung Park were involved in writing original draft, writing review and editing. Ae Ri Ahn conducted investigation and validation. Yong Chul Lee and Jae Seok Jeong were involved in writing original draft, reviewing, and the final approval of the manuscript and served as supervisors throughout the manuscript writing process.

## CONFLICT OF INTEREST STATEMENT

None declared.

## ETHICS STATEMENT

The authors declare that appropriate written informed consent was obtained for the publication of this manuscript and accompanying images.

## Data Availability

The data that support the findings of this study are available from the corresponding author upon reasonable request.
